# A recombinant spike protein subunit vaccine confers protective immunity against SARS-CoV-2 infection and transmission in hamsters

**DOI:** 10.1126/scitranslmed.abg1143

**Published:** 2021-08-11

**Authors:** Yangtao Wu, Xiaofen Huang, Lunzhi Yuan, Shaojuan Wang, Yali Zhang, Hualong Xiong, Rirong Chen, Jian Ma, Ruoyao Qi, Meifeng Nie, Jingjing Xu, Zhigang Zhang, Liqiang Chen, Min Wei, Ming Zhou, Minping Cai, Yang Shi, Liang Zhang, Huan Yu, Junping Hong, Zikang Wang, Yunda Hong, Mingxi Yue, Zonglin Li, Dabing Chen, Qingbing Zheng, Shaowei Li, Yixin Chen, Tong Cheng, Jun Zhang, Tianying Zhang, Huachen Zhu, Qinjian Zhao, Quan Yuan, Yi Guan, Ningshao Xia

**Affiliations:** 1 State Key Laboratory of Molecular Vaccinology and Molecular Diagnostics, National Institute of Diagnostics and Vaccine Development in Infectious Diseases, School of Life Sciences & School of Public Health, Xiamen University, Xiamen 361102, P. R. China.; 2 State Key Laboratory of Emerging Infectious Diseases, University of Hong Kong, Hong Kong 999077, P. R. China.; 3 Joint Institute of Virology (Shantou University and University of Hong Kong), Guangdong-Hongkong Joint Laboratory of Emerging Infectious Diseases, Shantou University, Shantou 515063, P. R. China.; 4 Research Unit of Frontier Technology of Structural Vaccinology, Chinese Academy of Medical Sciences, Xiamen 361102, Fujian, China.

## Abstract

Multiple safe and effective vaccines that elicit immune responses against severe acute respiratory syndrome coronavirus 2 (SARS-CoV-2) are necessary to respond to the ongoing coronavirus disease 2019 (COVID-19) pandemic. Here, we developed a protein subunit vaccine composed of spike ectodomain protein (StriFK) plus a nitrogen bisphosphonate–modified zinc-aluminum hybrid adjuvant (FH002C). StriFK-FH002C generated substantially higher neutralizing antibody titers in mice, hamsters, and cynomolgus monkeys than those observed in plasma isolated from COVID-19 convalescent individuals. StriFK-FH002C also induced both T_H_1- and T_H_2-polarized helper T cell responses in mice. In hamsters, StriFK-FH002C immunization protected animals against SARS-CoV-2 challenge, as shown by the absence of virus-induced weight loss, fewer symptoms of disease, and reduced lung pathology. Vaccination of hamsters with StriFK-FH002C also reduced within-cage virus transmission to unvaccinated, cohoused hamsters. In summary, StriFK-FH002C represents an effective, protein subunit–based SARS-CoV-2 vaccine candidate.

## Tackling transmission

An important feature of vaccines against severe acute respiratory syndrome coronavirus 2 (SARS-CoV-2) is whether or not the vaccine prevents transmission to others. In this study, Wu *et al.* vaccinated mice, hamsters, and cynomolgus monkeys with a SARS-CoV-2 spike protein subunit vaccine, StriFK, plus a nitrogen bisphosphonate–modified zinc-aluminum hybrid adjuvant called FH002C. The vaccine elicited antibody and cell-mediated immunity in all three models. StriFK-FH002C vaccination prevented hamsters from transmitting virus to unvaccinated, cohoused hamsters. This was associated with lower viral load in the upper respiratory tract of vaccinated hamsters after challenge. Thus, StriFK-FH002C represents an effective vaccine candidate for SARS-CoV-2.

## Teaser

A SARS-CoV-2 spike protein subunit vaccine induced humoral and cellular immune responses that protected hamsters against infection and prevented transmission.

## INTRODUCTION

The coronavirus pandemic caused by severe acute respiratory syndrome coronavirus 2 (SARS-CoV-2) is changing the landscape of global public health. To date, 218 countries and regions around the world have confirmed SARS-CoV-2 infections, with more than 181 million confirmed COVID-19 cases and nearly 4 million deaths. Although many efforts have slowed virus transmission, one of the essential tools to respond to the coronavirus disease 2019 (COVID-19) pandemic is an effective and safe vaccine.

The cellular entry of SARS-CoV-2 is predominantly mediated by the interaction of viral spike protein and its major receptor, the host angiotensin-converting enzyme 2 (ACE2) (*1*–*3*). Like other coronaviruses, the SARS-CoV-2 spike is a homotrimer protruding from the viral surface, and it comprises two functional subunits, namely, the S1 subunit containing the receptor binding domain (RBD) and the S2 subunit mediating membrane fusion between the virus and the cell (*4*–*6*). To date, several lines of evidence showed that antibodies against this spike protein could play a critical role in both prophylaxis against and therapeutics for COVID-19 (*7*–*12*). An array of distinct platforms is currently being used for vaccine development against COVID-19 (*13*–*19*). Subunit vaccines, particularly those that can be prepared using recombinant DNA technology, are advantageous because of their documented safety and compatibility with multiple boosts if necessary (*20*).

Two challenges, however, must be addressed when developing SARS-CoV-2 subunit vaccines. The first is to prepare the recombinant protein subunits in a functional form that can predominantly elicit SARS-CoV-2–neutralizing antibodies rather than nonneutralizing antibodies. This addresses concerns that nonneutralizing antibodies may mediate antibody-dependent enhancement (ADE) of disease or infection (*21*). The second is to overcome the relatively poor immunogenicity of subunit vaccines relative to intact virus vaccines or virus-like particle-based vaccines (*20*). Adding an adjuvant presents a solution to this challenge. Because the classical alum adjuvant generally stimulates T_H_2-biased immune response, its use could be associated with vaccine-associated enhanced respiratory disease (VAERD) (*22*). Thus, any adjuvant used should help to both generate a more balanced T_H_1 and T_H_2 cellular response and improve the immunogenicity of the subunit vaccine.

In this study, through in vivo evaluation of the immunogenicity of Chinese hamster ovary (CHO) cell–expressed various spike derivatives, we identified the full length of the spike ectodomain (StriFK) as the best performing immunogen because StriFK induced more neutralizing antibodies relative to nonneutralizing antibodies. In mice, hamsters, and nonhuman primates, we tested the immunostimulatory effects of different adjuvants with StriFK, specifically FH002C, a nitrogen bisphosphonate–modified zinc-aluminum hybrid adjuvant, and Al001, a traditional alum adjuvant commonly used in human vaccines.

## RESULTS

### StriFK induces spike-specific, neutralizing antibody responses in mice

**Fig. 1.**
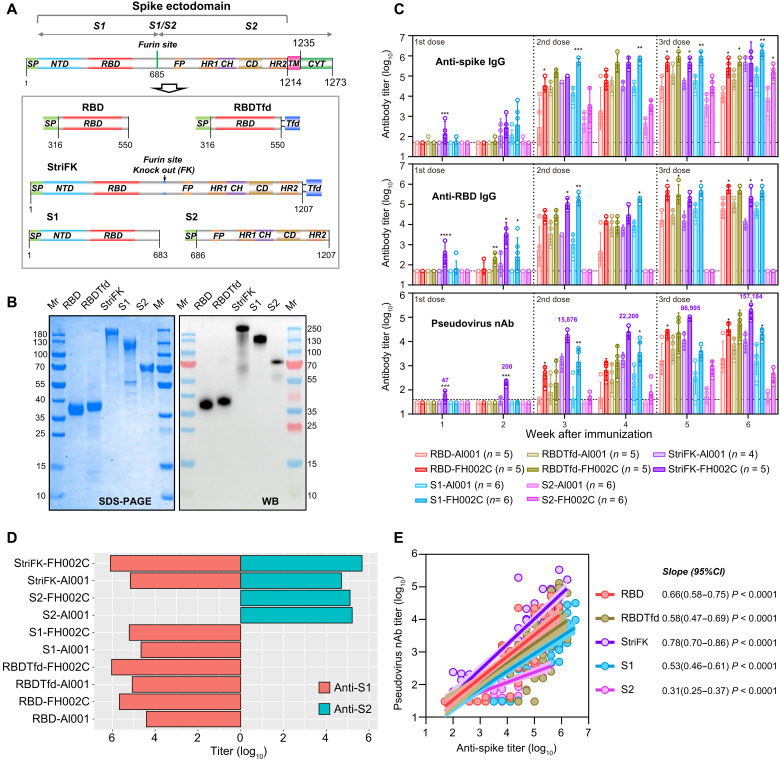
Immunogenicity of recombinant protein-based vaccine candidates was evaluated in mice. (**A**) Schematics of constructs for different recombinant spike derivatives. Functional domains of the SARS-CoV-2 spike are colored. NTD, N-terminal domain; RBD, receptor binding domain; FP, fusion peptide; HR1/2, heptad repeat 1/2; CH, central helix; TM, transmembrane domain; CYT, cytoplasmic tail; Tfd, trimerization motif of T4 fibritin (foldon). The borders for the spike functional domain were defined via amino acid sequence alignments of SARS-CoV-2 with SARS-CoV-1 and other human coronavirus, in accordance with previous reports (*2*, *53*). (**B**) SDS–polyacrylamide gel electrophoresis (PAGE) and Western blot (WB) analyses for purified proteins from CHO cells. Mr, protein marker; RBDTfd, RBD with C-terminal–fused Tfd; StriFK, furin site removed spike ectodomain with C-terminal–fused Tfd. A convalescent serum sample from a patient with COVID-19 was used for WB assay. (**C**) Antibody response induced by vaccine candidates in mice was measured. Groups of BALB/c mice were immunized with different vaccine candidates (1 μg per dose) at weeks 0, 2, and 4. The proteins of RBD, RBDTfd, StriFK, S1, and S2 were used as the immunogen combined with Al001 or FH002C adjuvants. Serum titers of anti-spike (top), anti-RBD (middle), and SARS-CoV-2 pseudovirus–neutralizing antibody (bottom) are displayed. Data were plotted as the geometric means with 95% confidence interval (CI). (**D**) Serum titers of anti-S1 and anti-S2 in immunized mice at week 6 after initial immunization. Data were plotted as the geometric mean. (**E**) Associations between the titers of pseudovirus nAb (neutralizing antibody) and anti-spike binding antibody in mice that received different immunogens. Data are derived from (C) and include sera from weeks 1 to 6. A Pearson test and linear regression model were used for correlation analysis. The linear regression curves with 95% CI were plotted. The slopes of regression curves were indicated to reflect the relative ratio of neutralizing-to-binding antibody induced by various immunogens. A Kruskal-Wallis test was used for intergroup statistical comparison in (C). Asterisks indicate statistical significance: ****P < 0.0001, ***P < 0.001, **P < 0.01, *P < 0.05.

Using a CHO expression system, recombinant spike protein derivatives of SARS-CoV-2, including RBD, S1, S2, trimerized RBD (RBDTfd), and StriFK, were obtained (Fig. 1A). All five purified proteins achieved acceptable purity and reacted in Western blots with antibodies present in the plasma of a patient after convalescence from COVID-19 (Fig. 1B). We combined StriFK with either alum (Al001) or FH002C as an adjuvant after confirming that both adjuvants showed similar morphology and antigen adsorption capacity (fig. S1). Among all tested candidates, the StriFK-FH002C–immunized mice presented the most rapid antibody response, showing detectable anti-spike, anti-RBD, and neutralizing antibodies just 1 week after administration of the first dose (Fig. 1C). Two weeks after the first dose, mice receiving StriFK or S1 (with either adjuvant) all presented detectable anti-spike antibodies. Likewise, mice that received RBDTfd-FH002C, StriFK-Al001, StriFK-FH002C, and S1-FH002C showed detectable anti-RBD antibodies (Fig. 1C, middle). However, at this time point, only mice immunized with StriFK-FH002C produced pseudovirus neutralizing antibody (Fig. 1C, bottom). Compared to Al001, FH002C adjuvant stimulated higher and more rapid antibody response in mice, regardless of the immunogen used. At week 6, after three-dose immunization was completed, the RBD, RBDTfd, S1, and StriFK induced similarly high titers of anti-spike and anti-RBD in combination with FH002C adjuvant.

As expected, anti-S2 antibodies were only observed at comparable titers in mice immunized with StriFK and S2 (Fig. 1D). Sera from S2-immunized mice showed detectable neutralizing antibodies, suggesting that both S1- and S2-specific antibodies contribute to the neutralization activity of StriFK-induced antibodies. Regression analyses on the associations between the anti-spike binding titer and the neutralizing titer in mice receiving various immunogens revealed that StriFK had a higher neutralizing-to-binding ratio (as reflected by a larger slope value) than the other antigens (Fig. 1E).

In a stable pool of CHO cells transfected with StriFK-expressing construct, protein expression reached more than 200 mg/liter (fig. S2A). The molecular weight of StriFK was determined to be about 700 kDa by high-performance liquid chromatography–size exclusion chromatography analysis (fig. S2B). StriFK showed that a binding affinity of 3.52 nM to human ACE2 protein (fig. S2C) is in line with previous studies (*2*, *6*). StriFK also reacted to human COVID-19 convalescent plasma samples in enzyme-linked immunosorbent assay (ELISA)–binding assay (fig. S2D). The StriFK-binding activity of convalescent plasma positively correlated with neutralizing titers against pseudotyped and authentic SARS-CoV-2 virus and was associated with their receptor-blocking titers (fig. S2E). Together, these findings support further evaluation of StriFK combined with the FH002C adjuvant as a vaccine candidate.

### StriFK-FH002C stimulates SARS-CoV-2–specific T cell responses in mice

**Fig. 2.**
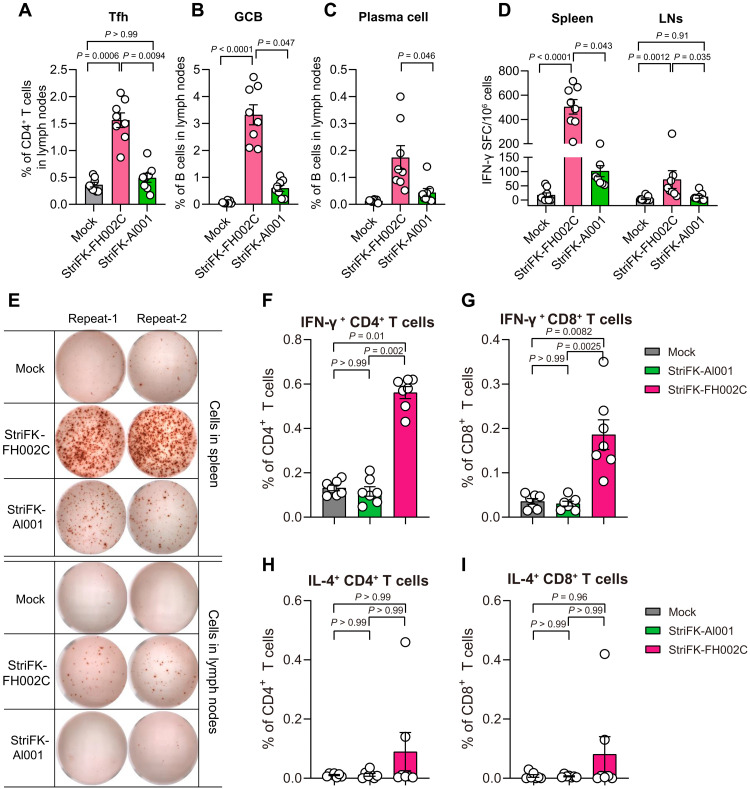
The StriFK-FH002C vaccine elicited potent humoral and cellular immune responses against the SARS-CoV-2 spike in mice. C57BL/6 mice were immunized with StriFK-FH002C and StriFK-Al001 (both at 10 μg per dose) on day 0 for ICS (*n* = 7 per group) or on days 0 and 21 for IFN-γ ELISPOT (*n* = 8 per group). Spleens and lymph nodes were collected at day 7 after the last immunization. (**A** to **C**) Percentages of lymph node follicular helper T (T_fh_) cells (A), germinal center B (GCB) cells (B), and plasma cells (C) that were induced by StriFK-FH002C and StriFK-Al001 for single-dose immunization. (**D**) The numbers of IFN-γ spot-forming cells (SFCs) isolated from the spleen and lymph node were quantified after stimulation of a peptide pool covering the entire spike protein. (**E**) Representative images of ELISPOT wells. (**F** to **I**) The frequency of IFN-γ^+^CD4^+^ T cells of total CD4^+^ T cells (F), IFN-γ^+^CD8^+^ T cells of total CD8^+^ T cells (G), IL-4^+^CD4^+^ T cells of total CD4^+^ T cells (H), and IL-4^+^CD8^+^ T cells of total CD8^+^ T cells (I) was determined by ICS after spike peptide pool stimulation. Data are presented as means ± SEM. The difference between the groups was analyzed by Kruskal-Wallis test and corrected for multiple comparisons using Dunn's method.

To explore the difference between FH002C and Al001 in inducing cellular immune response, we vaccinated C57BL/6 mice, which induce more robust T_H_1-skewed immune responses than BALB/c mice, with StriFK plus one of the two adjuvants. First, to test the effect of vaccination in promoting germinal center formation, we measured the frequencies of T follicular helper (T_fh_), germinal center B (GCB), and plasma cells in lymph nodes of mice 1 week after a single immunization (fig. S3). In comparison to StriFK-Al001, StriFK-FH002C significantly increased the frequencies of T_fh_ (*P* = 0.0094; Fig. 2A), GCB (*P* = 0.047; Fig. 2B), and plasma cells (*P* = 0.046; Fig. 2C), which were consistent with the more rapid antibody response and earlier antibody affinity maturation [indicated by higher immunoglobulin G (IgG) avidity] observed after StriFK-FH002C immunization (fig. S4A). Sera from StriFK-FH002C–immunized animals showed significantly higher IgG2a (*P* = 0.032) or IgG2b (*P* = 0.016) titers (fig. S4B) and higher IgG2b-to-IgG1 titer ratio (fig. S4C) than StriFK-Al001. Together, these results demonstrated that StriFK-FH002C induced more balanced T_H_1 and T_H_2 immune responses.

In mice receiving two immunizations of StriFK-FH002C or StriFK-Al001, we further measured spike-specific T cell responses. In contrast to nonimmunized animals, interferon-γ (IFN-γ) enzyme-linked immunosorbent spot (ELISPOT) assays revealed the numbers of IFN-γ–secreting cells after ex vivo antigen-peptide stimulation increased by 28.9-fold and 5.8-fold in the spleen and 14.0-fold and 2.3-fold in lymph nodes in mice immunized with StriFK-FH002C or StriFK-Al001, respectively (Fig. 2, D and E). Compared to Al001, the FH002C adjuvant elicited significantly higher frequencies of spike-specific IFN-γ–secreting cells in either spleen (*P* = 0.043) or lymph nodes (*P* = 0.035).

Intracellular cytokine staining (ICS) measurements of mouse splenocytes stimulated by spike peptides demonstrated that StriFK-FH002C induced stronger T_H_1 and cytotoxic T lymphocyte (CTL) responses than StriFK-Al001, which was shown by significantly higher frequencies of spike-specific IFN-γ^+^CD4^+^ (*P* = 0.002; Fig. 2F) and IFN-γ^+^CD8^+^(*P* = 0.0025; Fig. 2G and fig. S5). There was no difference in the percentage of interleukin-4 (IL-4)^+^CD4^+^ T cells and IL-4^+^CD8^+^ T cells between the two groups (*P* > 0.99; Fig. 2, H and I). Therefore, compared with Al001 adjuvant, FH002C adjuvant can induce a stronger T_H_1 cell response.

### StriFK-FH002C is immunogenic in nonhuman primates and hamsters

Next, we examined the immunogenicity of StriFK-FH002C in cynomolgus macaques in comparison to StriFK-Al001 and RBD-FH002C (fig. S6). At week 4, all animals receiving the two-dose regimen showed detectable anti-spike (fig. S6A), neutralizing (fig. S6B), and receptor-blocking antibodies (fig. S6C). In contrast to RBD, StriFK stimulated higher antibody titers in all three assays, particularly for the first and second dose. On the other hand, the results after one dose imply that the FH002C adjuvant may confer an advantage in accelerating antibody generation. After three immunizations, animals immunized with each of the three vaccine candidates displayed similarly high titers of neutralizing antibodies and receptor-blocking antibodies, suggesting that the advantages of StriFK-FH002C were mainly reflected in the rapid antibody response in the early immunization phase. However, the highest neutralizing response was still observed in monkeys that received three-dose StriFK-FH002C immunization at week 7 (fig. S6B). No abnormalities in body weight (fig. S6D), body temperature (fig. S6D), liver function (fig. S6E), renal function (fig. S6F), cardiac enzymes (fig. S6G), or other clinical manifestations were observed in StriFK-FH002C– or StriFK-Al001–immunized animals, suggesting a good safety profile of these vaccines in nonhuman primates.

**Fig. 3.**
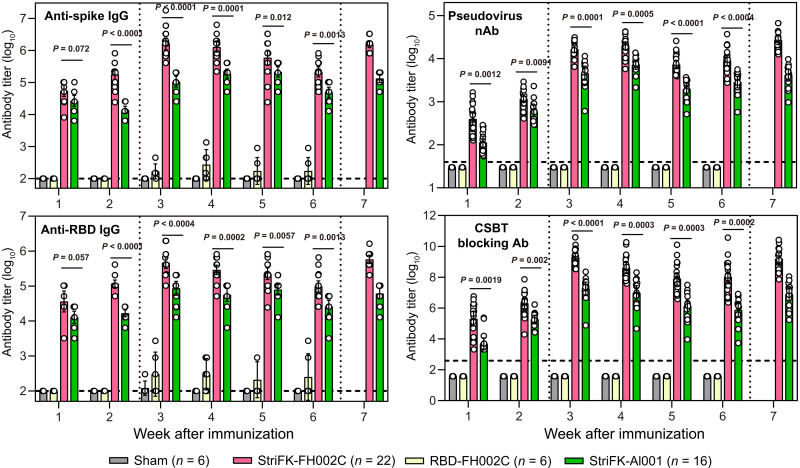
Antibody response were induced in hamsters after immunization with StriFK-FH002C, RBD-FH002C, or StriFK-Al001. All three vaccines were used at 10 μg per dose. Animals in sham (FH002C-only) and RBD-FH002C groups received two doses of vaccine at weeks 0 and 2, whereas animals in the StriFK-Al001 group received three doses of vaccine at weeks 0, 2, and 6. In the StriFK-FH002C group, 6 of 22 animals received two-dose immunizations at weeks 0 and 2, and the remaining 16 animals received three-dose immunizations at weeks 0, 2, and 6. Serum samples were collected every week after initial immunization, and the anti-spike, anti-RBD, neutralizing antibody, and the receptor-blocking antibody titers were measured. Receptor-blocking titers were quantified by CSBT. Antibody titers are shown as geometric means with 95% CI. A Kruskal-Wallis test was used for intergroup statistical comparison and corrected for multiple comparisons used Dunn's method.

Nonhuman primates have been reported as a mild disease model for SARS-CoV-2 infection (*23*), whereas hamsters are considered suitable animal models mimicking severe COVID-19 pneumonia in humans (*24*–*26*). We next assessed the immunogenicity of vaccine candidates in Syrian golden hamsters (*Mesocricetus auratus*), aiming to evaluate protective efficacy of candidate vaccines using this model (Fig. 3). Hamsters that received StriFK-based vaccines (either StriFK-FH002C or StriFK-Al001) quickly generated detectable antibodies as early as week 1 after the first vaccine in all four assays measured. At week 3, or 7 days after the second vaccine dose, StriFK-FH002C elicited the highest titers of anti-RBD antibodies [geometric mean titer (GMT) = 462,178; range: 102,400 to 1,968,300), anti-spike antibodies (GMT = 1,604,827; range: 409,600 to 17,714,700), neutralizing antibodies (GMT = 16,617; range: 6478 to 31,220), and receptor-blocking antibodies (GMT = 634; range: 369 to 1537; Fig. 3). From weeks 3 to 6, the antibody titers in StriFK-FH002C– or StriFK-Al001–immunized animals slightly decreased to about 50% of the corresponding peak values. When a booster immunization was given at week 6, the serum antibodies of animals in groups StriFK-FH002C and StriFK-Al001 rapidly increased to comparable concentrations as those observed at week 3. In hamsters, StriFK-FH002C induced more than fourfold higher neutralizing receptor-blocking antibody titers than StriFK-Al001, suggesting a potent immunostimulatory effect of the FH002C adjuvant (Fig. 3). RBD-FH002C, which could generate binding and functional antibodies in mice and monkeys, produced minimal antibody responses in hamsters.

### StriFK-FH002C protects against SARS-CoV-2 infection and pathogenicity in hamsters

We investigated the protective efficacy of StriFK-FH002C in hamsters using intranasal SARS-CoV-2 challenge. We first tested whether a two-dose immunization of StriFK-FH002C could protect hamsters from SARS-CoV-2 infection and pneumonia. In this experiment, the neutralizing antibody GMT of the six StriFK-FH002C–vaccinated hamsters was 14,404 (range: 5368 to 37,078). In the sham group that received FH002C only, hamsters lost an average of 8.4, 12.2, and 11.7% of body weight by 3, 5, and 7 days postinfection (dpi), respectively (fig. S7A). In contrast, StriFK-FH002C–immunized animals lost just an average of 2.0, 2.1, and 1.2% of body weight by the corresponding time points (fig. S7A), which was significantly lower than that of the sham controls (*P* < 0.0001) and similar to that of the uninfected (mock) group (*P* = 0.061). All animals in the sham group exhibited symptoms of weakness, piloerection, hunched back, or abdominal respiration, which were not observed in vaccinated or uninfected animals (fig. S7B).

Animals immunized with two doses of vaccine in sham and vaccine groups were euthanized at 3, 5, or 7 dpi (two animals per time point) to assess viral loads and histopathological profiles in the respiratory tract. For sham controls, the peak viral loads in lung tissues were detected at 3 dpi [6.4 ± 0.3 log_10_ plaque-forming units (PFU)/ml] and gradually decreased at 5 dpi (5.4 ± 0.3 log_10_ PFU/ml) and 7 dpi (2.5 ± 0.3 log_10_ PFU/ml). In the StriFK-FH002C group, infectious virus was only detected in lung tissues of hamster FV002 (4.2 log_10_ PFU/ml) at 3 dpi but not in tissues of other animals (fig. S7C). The relatively lower neutralizing antibody titer in FV002 [median inhibitory dilution (ID_50_) = 5368] than the others (ID_50_ range: 9363 to 37,078) was a possible explanation for the insufficient protection of this animal individual. However, the lung viral titer of the FV002 hamster remained markedly lower than those from sham controls. The nucleocapsid immunostainings in the lungs were largely consistent with viral titer profiles (fig. S7D). Pathological examination of the lung sections by hematoxylin and eosin (H&E) staining demonstrated typical features of moderate-to-severe interstitial pneumonia in sham controls (fig. S7E). In contrast, vaccinated animals only showed slight histological changes with reduced pathological severity (fig. S7E).

**Fig. 4.**
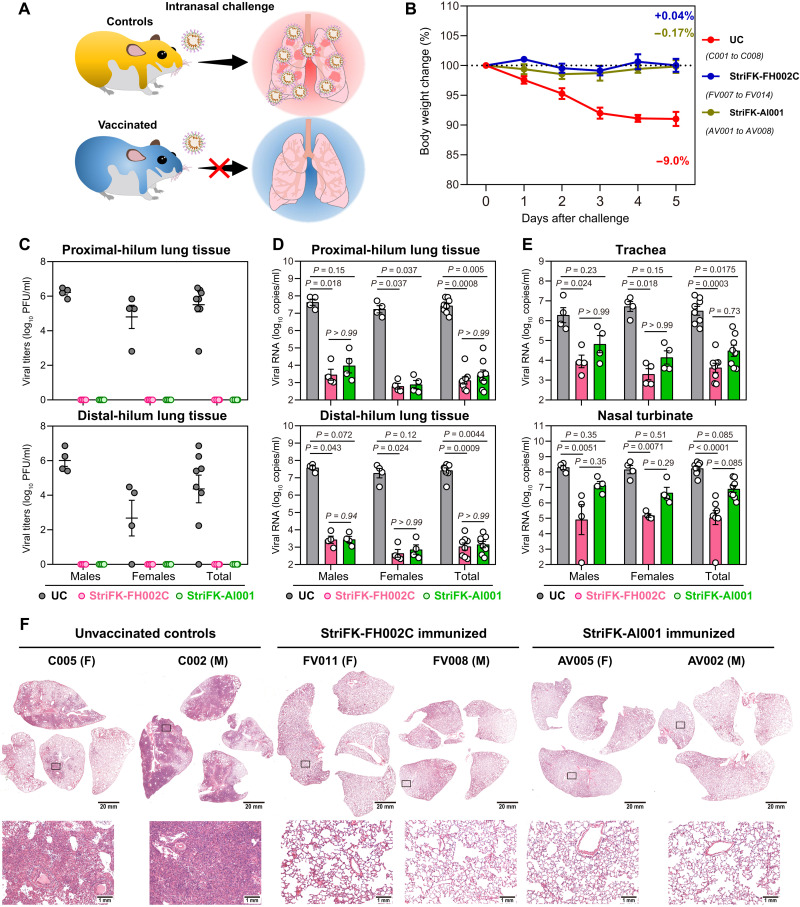
Three immunizations with StriFK-FH002C and StriFK-Al001 protect against intranasal SARS-CoV-2 challenge in hamsters. (**A**) Schematic of the direct virus challenge study. A total of 24 hamsters (half males and half females), including 8 unvaccinated animals [unvaccinated controls (UC), C001 to C008], 8 animals vaccinated three times with StriFK-FH002C (FV007 to FV014), and 8 animals vaccinated three times with StriFK-Al001 (AV001 to AV008), were intranasally challenged with 1 × 10^4^ PFU of SARS-CoV-2. All animals were euthanized for tissue analyses at 5 dpi. (**B**) Changes in body weight after virus challenge were plotted. The average weight loss of each group at 5 dpi is indicated as a colored number. Data are means ± SEM. (**C**) Infectious viral titers were quantified in lung regions proximal (top) or distal (bottom) to the pulmonary hilum. (**D**) Viral RNA concentrations from lung regions proximal (top) or distal (bottom) to the pulmonary hilum were quantified. (**E**) Viral RNA concentrations in lysates of the trachea (top) and nasal turbinate (bottom) from hamsters were quantified. Data are means ± SEM. The difference between the groups was analyzed by Kruskal-Wallis test and corrected for multiple comparisons using Dunn's method. (**F**) H&E-stained lung sections collected from virus-challenged hamsters. Views of the whole lung lobes (four independent sections) are presented in the top panel (scale bars, 20 mm), and the areas in the black box were enlarged in the bottom panel (scale bars, 1 mm).

To further evaluate the protective efficacy of StriFK-FH002C and to reveal the role of FH002C adjuvant, hamsters were immunized three times with StriFK-FH002C or StriFK-Al001 (*n* = 8 each) and subsequently challenged with SARS-CoV-2 (Fig. 4A). The neutralizing and receptor-blocking antibody titers in StriFK-FH002C vaccinated animals were about fourfold higher than that in StriFK-Al001–vaccinated animals (Fig. 3). The average weight loss at 5 dpi was 9.0, −0.04, and 0.17% in the unvaccinated controls, the StriFK-FH002C group, and the StriFK-Al001 group, respectively (Fig. 4B). The weight loss was markedly lower in both vaccinated groups compared to unvaccinated controls (*P* < 0.0001 for both comparisons) and showed no difference between the StriFK-FH002C and StriFK-Al001 groups (*P* = 0.39). Infectious virus was detected in the lungs of all unvaccinated animals. In the lung tissues of vaccinated hamsters, no infectious virus was detected (Fig. 4C).

Consistent with infectious virus titers, viral RNA concentrations were profoundly reduced in lung tissue from vaccinated hamsters compared to the controls (Fig. 4D). In trachea and nasal turbinate tissues, StriFK-FH002C– and StriFK-Al001–immunized hamsters exhibited a larger reduction in viral RNA relative to controls (Fig. 4E). No differences were observed between sexes for vaccination-mediated protection. Gross lung pictures demonstrated multifocal diffuse hyperemia and consolidation in the lungs of controls, and the apparent lesions were markedly diminished in both vaccinated groups (fig. S8). Histopathological examinations observed pulmonary consolidation, alveolar destruction, and diffuse inflammation in about 20 to 30% of lungs from female controls and 50 to 60% of lungs from male controls. In comparison to controls, vaccinated animals only showed mild inflammation in limited areas of lungs and had minimal pneumonia pathology in most areas of the lung (Fig. 4F). The pathological severity scores of vaccinated hamsters were slightly higher than that of unchallenged animals (fig. S9, A and B) but were significantly lower than virus-infected controls (*P* < 0.05 for both vaccine groups; fig. S9B). These results demonstrated that StriFK-FH002C immunization protected hamsters from SARS-CoV-2 virus infection and pathology.

### StriFK-FH002C reduces transmission of SARS-CoV-2 between cohoused hamsters

**Fig. 5.**
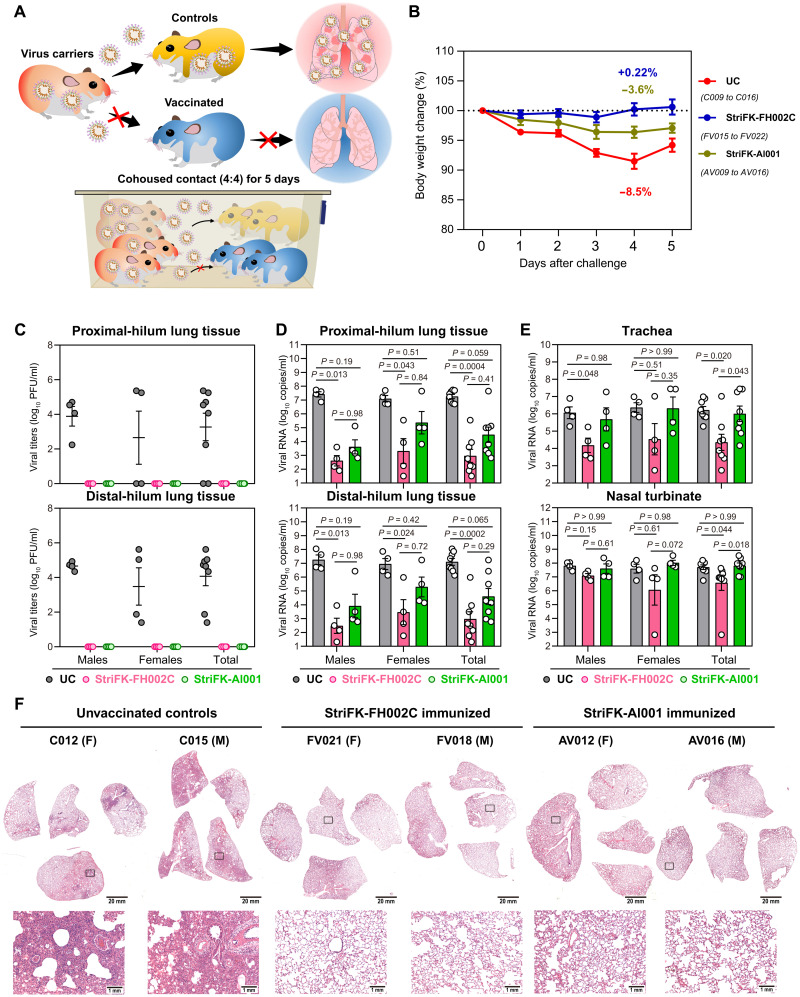
Three immunizations with StriFK-FH002C and StriFK-Al001 reduces SARS-CoV-2 transmission in cohoused hamsters. (**A**) Schematic of virus transmission study. First, 24 hamsters were preinfected with 1 × 10^4^ PFU of SARS-CoV-2 as virus donors. One day later, four donors were cohoused with four vaccinated or control hamsters for a 5-day follow-up. A total of 24 hamsters (male/female = 1:1), including 8 unvaccinated animals (UC, C009 to C016) and 16 immunized animals, which received either three doses of StriFK-FH002C (*n* = 8, FV015 to FV022) or StriFK-Al001 (*n* = 8, AV009 to AV016), were exposed to SARS-CoV-2–infected donors. (**B**) Changes in body weight after cohoused exposure. The average weight loss of each group at 4 dpi (the time point that controls showed maximum percent weight loss) is indicated as a colored number. Data are means ± SEM. (**C**) Infectious viral titers were quantified from lung tissue proximal (top) or distal (bottom) to the pulmonary hilum. (**D**) Viral RNA concentrations are shown for lung tissue proximal (top) or distal (bottom) to the pulmonary hilum. (**E**) Viral RNA concentrations in lysates of the trachea (top) and nasal turbinate (bottom) tissues from hamsters. Data are means ± SEM. The difference between the groups was analyzed by Kruskal-Wallis test and corrected for multiple comparisons using Dunn's method. (**F**) H&E-stained lung sections collected from cohoused hamsters. Views of the whole lung lobes (four independent sections) are presented in the top panel (scale bars, 20 mm), and the areas in the black box were enlarged in the bottom panel (scale bars, 1 mm).

We next assessed whether StriFK-FH002C could protect against SARS-CoV-2 transmission in hamsters (Fig. 5A). Unvaccinated control hamsters cohoused with infected hamsters exhibited peak weight loss on day 4 after contact exposure (Fig. 5B). In contrast, weight loss was diminished in both vaccinated groups (StriFK-Al001 versus controls, *P* = 0.0091; StriFK-FH002C versus controls, *P* < 0.0001), and it changed to a lesser degree (*P* = 0.017) in the StriFK-FH002C group than in the StriFK-Al001 group. None of the vaccinated animals had detectable infectious virus in their lungs at day 5 after exposure, in contrast to control animals (Fig. 5C). Furthermore, vaccinated hamsters had lower viral RNA concentrations in the lung, trachea, and nasal turbinate compared to unvaccinated controls (Fig. 5, D and E). Gross lung images (fig. S10) and pathological analyses (Fig. 5F) indicated that control hamsters had extensive lung damage with consolidated lesion and inflammatory cell infiltration across larger areas, which was similar to that observed in hamsters directly challenged with intranasal SARS-CoV-2. In contrast, both vaccines prevented tissue damage to a large degree, with mild perivascular and alveolar infiltration observed in very few areas (Fig. 5F). There was no difference between the lung pathological severity scores of vaccinated hamsters and unchallenged animals (*P* > 0.99 for both vaccine groups), suggesting efficient protection of vaccination from SARS-CoV-2–induced lung pathology (fig. S9B).

**Fig. 6.**
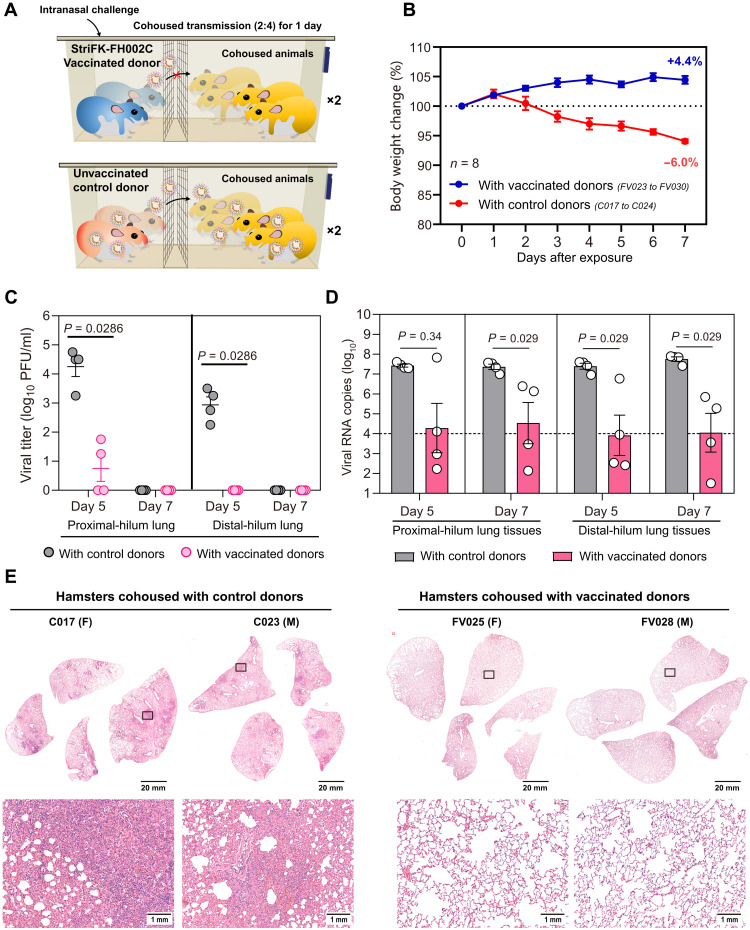
StriFK-FH002C reduces virus transmission from vaccinated donors to unvaccinated animals. (**A**) Schematic of the experimental design. Four StriFK-FH002C vaccinated hamsters (two males and two females) or four unvaccinated hamsters were intranasally challenged with 1 × 10^4^ PFU SARS-CoV-2 as virus donors. One day later, two vaccinated donors or two unvaccinated donors were cohoused with four unvaccinated hamsters (separated by a double-layer ventilated fence in the same cage). After 24 hours, the donors were removed and the 16 exposed hamsters (8 males and 8 females), including 8 cohoused with unvaccinated donors (C017 to C024) and 8 cohoused with vaccinated donors (FV023 to FV030), were followed for 7 days. On days 5 or 7 after cohousing, half of the animals were euthanized for tissue analyses, respectively. (**B**) Changes in body weight after cohoused exposure. Data are means ± SEM. (**C** and **D**) Infectious viral titers (C) and viral RNA concentrations (D) for lung tissue collected on day 5 or 7 after exposure. Data are means ± SEM. A Mann-Whitney *U* test was used for intergroup statistical comparison. (**E**) H&E-stained lung sections collected from cohoused hamsters. Views of the whole lung lobes (four independent sections) are presented in the top panel (scale bars, 20 mm), and the areas in the black box were enlarged in the bottom panel (scale bars, 1 mm).

The potential for StriFK-FH002C vaccination to reduce virus transmission from vaccinated, subsequently infected donors to unvaccinated controls was also evaluated. In this experiment, both StriFK-FH002C–vaccinated and unvaccinated control hamsters were challenged with intranasal SARS-CoV-2 to serve as potential virus donors. One day later, virus-challenged animals were cohoused with unvaccinated hamsters separated by a double-layer ventilated fence in the same cage (Fig. 6A). Hamsters cohoused with unvaccinated donors exhibited maximal weight loss on day 7 after exposure, whereas the hamsters cohoused with vaccinated donors exhibited increased weight (Fig. 6B). On day 5 after exposure, infectious virus was detected in the lungs of all four animals that were cohoused with unvaccinated donors. In contrast, only 2 of 4 (50%) animals that were cohoused with vaccinated donors showed evidence of infectious virus in their proximal-hilum lung tissues (Fig. 6C). On day 7 after exposure, no infectious virus was detected in the lungs of animals in either group. Moreover, all hamsters cohoused with unvaccinated donors showed a high concentration (>10^7^ copies/ml) of viral RNA in their lung tissue on both days 5 and 7 after exposure. For hamsters cohoused with vaccinated donors, 4 of 8 (50%) exhibited low viral RNA concentrations (<10^4^ copies/ml) in the lungs (Fig. 6D). Further H&E-staining analyses revealed that the lungs from hamsters cohoused with unvaccinated donors had multifocal lung damage, whereas only minimal pathological lesions were observed in lung tissues from animals cohoused with vaccinated donors (Fig. 6E and fig. S9B). These results demonstrated that StriFK-FH002C vaccination not only could protect vaccinated animals from SARS-CoV-2 infection but also had an effect in reducing virus transmission from vaccinated to unvaccinated animals.

### The protective efficacy of StriFK-FH002C vaccination was correlated with neutralizing and receptor-blocking antibody titers

**Fig. 7.**
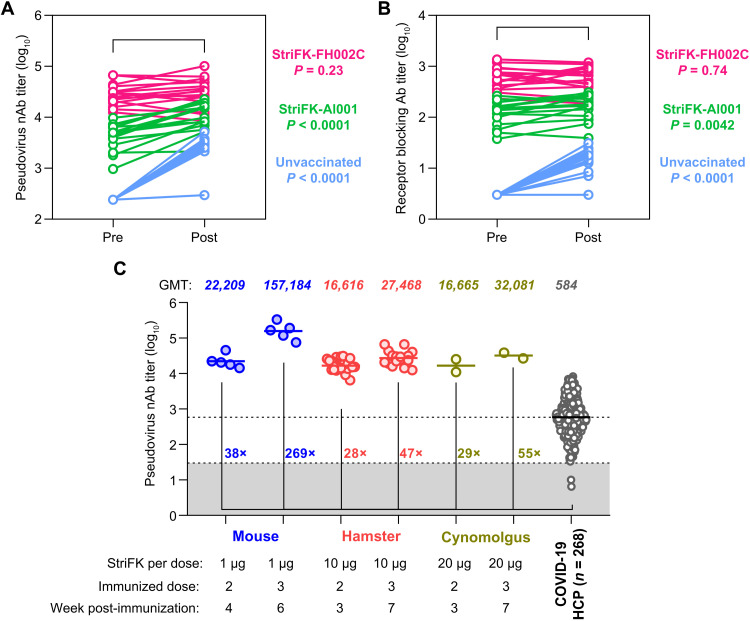
Vaccination with StriFK-FH002C elicited robust protective antibody titers and conferred sterilizing immunity against SARS-CoV-2 in animal models. (**A** and **B**) Comparison of the pseudovirus nAb (A) or receptor-blocking antibody (B) titers in paired sera of hamsters collected before (Pre) and 5 days (Post) after SARS-CoV-2 challenge. Samples from the same hamster are connected with a line. Vaccinated hamsters and unvaccinated controls were pooled for analysis. A paired Wilcoxon signed-rank test was used for statistical analysis. (**C**) Pseudovirus neutralizing antibody titers in human convalescent plasma (HCP) samples from individuals diagnosed with COVID-19 were compared to those from StriFK-FH002C vaccinated animals. A total of 268 convalescent plasma samples were collected from patients who had recovered from COVID-19 within 8 weeks since illness onset. The GMT of pseudovirus nAb is shown for COVID-19 HCP and for StriFK-FH002C vaccinated mice (*n* = 6), hamsters (*n* = 22), and cynomolgus macaques (*n* = 2).

Using pooled samples (Figs. 4 and 5), we analyzed associations between StriFK-elicited functional antibody titers and indices for protective efficacy. We demonstrated that the serum titers (before the virus challenge) of neutralizing antibody and receptor-blocking antibody were negatively correlated with viral RNA concentrations in tissues of nasal turbinate, trachea, and lung from virus-challenged hamsters (fig. S11). We also compared the serum antibody titers of hamsters before and after the SARS-CoV-2 challenge (Fig. 7, A and B). Control hamsters showed an average of 24-fold (*P* < 0.0001) and 5.4-fold (*P* < 0.0001) increase in neutralizing antibody and receptor-blocking antibody, whereas the StriFK-Al001–immunized animals presented only 2.8-fold (*P* < 0.0001) and 1.4-fold (*P* = 0.0042) elevation in corresponding antibody markers. The StriFK-FH002C–immunized hamsters showed no significant increase in either neutralizing antibody (*P* = 0.23) or receptor-blocking antibody (*P* = 0.74) concentrations after SARS-CoV-2 challenge (Fig. 7, A and B). We next asked whether StriFK-FH002C or StriFK-Al001 vaccination induced anamnestic antibody responses, which are associated with less efficient neutralizing antibody responses. Evidence of anamnestic antibody responses can be shown by an increase in neutralizing antibody titers after infection. Using a twofold increase in neutralizing antibody titers as a cutoff (where a greater than twofold increase indicates the presence of an anamnestic response), we concluded that there was no evidence of an anamnestic response in 15 of 16 animals in the StriFK-FH002C–vaccinated group or in 6 of 16 animals in the StriFK-Al001–vaccinated group (Fig. 7A). We also measured the serological antibodies against SARS-CoV-2 nucleocapsid protein (NP) for these animals. Among vaccinated or control animals challenged directly with intranasal SARS-CoV-2, anti-NP seroconversion was observed in most serum pairs of tested animals (23 of 24; fig. S12A), which may be attributed to the direct immune stimulation derived from the free NP proteins in unpurified virus supernatants. In contrast, among animals involved in the contact transmission experiment, anti-NP seroconversion was observed in eight (100%), two (25%), and zero hamsters in the unvaccinated group, StriFK-Al001 group, and StriFK-FH002C group, respectively (fig. S12B). Overall, these data suggested that sterilizing immunity to SARS-CoV-2 may have been conferred by StriFK-FH002C immunization, at least in a subset of animals evaluated. The pooled antibody data in mice, hamsters, and nonhuman primates showed that the neutralizing antibodies induced by StriFK-FH002C in animals were about 30- to 250-fold higher than that of convalescent plasma isolated from humans diagnosed with COVID-19 (Fig. 7C).

## DISCUSSION

In this study, we systematically evaluated the immunogenicity and protective efficacy of a recombinant SARS-CoV-2 subunit vaccine candidate, StriFK-FH002C, in three animal models. This vaccine candidate was composed of CHO-expressed SARS-CoV-2 spike ectodomain protein (StriFK) with FH002C as an adjuvant. Comparing to other tested spike derivatives, including RBD, S1, and S2, our in vivo data demonstrated that StriFK generated a higher neutralizing-to-binding ratio, which implied that it induced more neutralizing antibodies than nonneutralizing antibodies. FH002C is a functionalized zinc-aluminum hybrid adjuvant with an immune-enhancing effect. Immunization with StriFK-FH002C in mice, hamsters, and monkeys rapidly generated anti-spike, neutralizing, and receptor-blocking antibodies after one administration. Two doses of StriFK-FH002C induced more than 30-fold higher neutralizing antibody titers as compared to titers of human COVID-19 convalescent plasma samples. Furthermore, three doses of StriFK-FH002C induced 50- to 250-fold higher neutralizing antibody titers than convalescent plasma samples.

In hamsters, an animal model recapitulating severe human COVID-19 disease, StriFK-FH002C vaccination provided protective immunity to prevent SARS-CoV-2 infection, pathogenesis, and transmission. The correlation between functional (neutralizing or receptor blocking) antibody titers and reduced SARS-CoV-2 virus load demonstrated protection mediated by this vaccine. It has been reported that anamnestic responses after virus challenge, which usually occurred in animals with inadequate neutralizing antibodies, can be a surrogate to evaluate whether a vaccination provides sterilizing immunity (*17*). A recent study reported that a single dose of live-attenuated YF17D-vectored SARS-CoV-2 vaccine could induce sterilizing immunity in at least a subset of vaccinated animals (4 of 12 vaccinated animals had no anamnestic neutralizing antibodies) (*27*). Anamnestic neutralizing antibody responses were not observed in 15 of 16 StriFK-FH002C–vaccinated animals or in 6 of 16 StriFK-Al001–vaccinated animals after SARS-CoV-2 challenge, suggesting that StriFK-FH002C immunizations may have afforded sterilizing immunity in at least a subset of hamsters. Other evidence to support this includes undetectable infectious virus and low viral RNA concentrations in the lungs of StriFK-FH002C–vaccinated animals. Our data demonstrated that StriFK-FH002C immunization reduced SARS-CoV-2 transmission from vaccinated to unvaccinated hamsters. A recently published study also suggested that the StriFK-FH002C vaccine candidate largely prevented SARS-CoV-2 replication and lung damage caused by SARS-CoV-2 infection in minks, which is another severe disease model (*28*). Although the protective efficacy of StriFK-FH002C in nonhuman primates was not tested here, the comparable titers of neutralizing and receptor-blocking antibodies induced by this vaccine in both models suggests that the vaccine would confer protection.

Apart from the highly immunogenic StriFK protein, the FH002C adjuvant also played an essential role in generating potent protective antibody and spike-specific T cell responses. Due to the multiple mechanisms by which adjuvants function, “adjuvant systems” comprising more than one component have been proposed (*29*). In FH002C, a drug for osteoporosis nitrogen bisphosphonate (risedronate) was loaded on the composite alum adjuvant by harnessing the alum’s phosphophilic nature. FH002C also incorporates zinc ions, which become available upon adjuvant dissolution. Zinc ions have an immunostimulatory effect and are known to skew the immune response toward the T_H_1 pathway (*30*, *31*). Although the precise role of nitrogen bisphosphonate is still unknown, previous studies suggested that these compounds could stimulate γδ T cell expansion and act as a dendritic cell modulator because of the upstream accumulation of phosphoantigen isopentyl diphosphate resulting from the inhibition of farnesyl diphosphate synthetase (*32*–*34*). The FH002C adjuvant showed more potent effects than Al001 in promoting the development of T_fh_ and GCB cells and showed generation of antibody-producing plasma cells in the early days after immunization. Because of the rapid differentiation of these cell populations, FH002C adjuvant–based StriFK vaccine was shown to generate a faster and stronger protective antibody response, which elicited neutralizing antibodies as early as 1 week after the initial immunization. This advantage enables the vaccine’s potential application for emergency vaccination of close contacts because of high-risk exposure, aiming to quickly control outbreaks among close contacts and in the naïve population.

In addition to neutralizing antibodies, T cells are also believed to be important for vaccine efficacy (*35*–*37*). The spike of SARS-CoV-2 has a subnanomolar affinity to its ACE2 receptor, and infections may occur at the respiratory epithelium of the upper respiratory tract, where antibody concentration is low. Moreover, antibodies may not completely block viruses from invading cells. Virus-specific T cells may thus play a role in eradicating virus-infected cells or inhibiting viral replication and spread via noncytotoxic effects (*38*, *39*). Our in vivo data showed that StriFK-FH002C immunization could induce spike-specific IFN-γ^+^CD8^+^ and IFN-γ^+^CD4^+^ T cell responses, which would help to establish protective immunity in vaccinated individuals.

Safety is also a crucial concern for COVID-19 vaccine development. Previous studies on respiratory syncytial virus and measles vaccine suggested that VAERD is linked to T_H_2-biased immune responses (*40*–*42*). A similar phenomenon was also noted in animal studies regarding SARS-CoV-1 vaccines (*40*). Therefore, an ideal COVID-19 vaccine is expected to elicit a balanced T_H_1 and T_H_2 immune response. Recombinant protein vaccines adjuvanted by traditional aluminum adjuvants generally induce enhanced T_H_2-biased immune responses but insufficient T_H_1 responses. We observed this in the performance of our StriFK-Al001 vaccine in the mouse model, as indicated by the lower IgG2-to-IgG1 titer ratio. The FH002C adjuvant may be a tool to overcome this drawback. Compared to traditional alum adjuvant (Al001) used in licensed hepatitis E virus (*43*) and human papillomavirus vaccines (*44*), FH002C potently elicited both virus-specific T_H_1 and T_H_2 responses, as shown by the simultaneous increase in IFN-γ^+^CD4^+^ and IL-4^+^CD4^+^ T cells in splenocytes of immunized mice. ICS and ELISPOT assays further demonstrated that StriFK-FH002C could elicit a more robust spike-specific CTL response than StriFK-Al001, which may result from an enhanced T_H_1 response. Because the hamster is a susceptible model of SARS-CoV-2 infection and pathogenesis (*24*, *25*), all StriFK-FH002C–vaccinated hamsters showed markedly diminished virus infection and disease severity without evidence of VAERD in our study. Although FH002C has not been tested in humans, risedronate, a component of FH002C, was clinically used to treat osteoporosis over 20 years ago (*45*). Each dose of risedronate in the adjuvant introduces less than ^1^/_10_ of what one would take in a daily oral dose (5 mg) (*45*). In the study, we did not find any systemic side effects in mice, hamsters, or nonhuman primates immunized with the FH002C-adjuvanted vaccine. These results suggest a good safety profile of StriFK-FH002C in animals and support further clinical development.

To date, more than 180 vaccine candidates based on various platforms are under development against SARS-CoV-2 (*19*). StriFK-FH002C differs from other reported subunit vaccine candidates in two aspects: the unique FH002C adjuvant and the non–2P-mutated StriFK immunogen. FH002C does not rely on natural products (such as squalene or QS-21), which may limit manufacturing capacity. Instead, it uses readily available, active pharmaceutical ingredients, making it more scalable and more sustainable during the outbreaks and long-term use. In terms of antigen design, most reported spike protein–based vaccines are 2P-mutated, but S-2P production in mammalian cells is difficult due to low expression after transient transfection (*2*, *46*). In our hands, StriFK production could reach a concentration of 40 to 60 mg/liter in transiently transfected CHO cells, and StriFK stably transfected cell pools expressed the protein as concentrations of greater than 200 mg/liter. A cloned high-production cell line produced more than 500 mg/liter. Our data also demonstrate StriFK’s promising immunogenicity and protection in animals. The high yield and simple production of StriFK may enable improved manufacturing and scalability for further applications. Using the CHO expression platform and FH002C, various spike proteins from different disease-causing coronavirus strains could be expressed ahead of time, enabling quick antigen preparation and vaccine formulation for rapid vaccine development against emerging strains of SARS-CoV-2. It is conceivable that this could be an effective approach for helping to control virus spread.

There are several limitations to our study. Although we have demonstrated the preliminary safety of StriFK-FH002C in cynomolgus monkeys, it is still necessary to conduct systematic toxicological studies on FH002C or StriFK-FH002C before clinical application. Moreover, the precise mechanism by which FH002C stimulates the immune response is not fully elucidated, and further exploration is needed. The correlation between vaccine-induced T cell responses and immune protection has not been determined in this study because of the lack of tools to study T cell responses in hamsters.

In summary, we developed a SARS-CoV-2 subunit vaccine based on recombinant StriFK protein and a functionalized adjuvant with unique immunostimulatory properties. Our evaluations demonstrated that StriFK-FH002C was well tolerated and exhibited good immunogenicity in mice, hamsters, and nonhuman primates. Vaccination with StriFK-FH002C provided effective protection against infection, pathogenesis, and transmission of SARS-CoV-2 in hamsters. This vaccine candidate is under further development in the preclinical stage and could be manufactured on a large scale, with the hope that a stronger and more sustainable immune response could be elicited using this vaccine as compared to natural infection.

## MATERIALS AND METHODS

### Study design

The primary objective of this study was to study the immune response and protective effects of StriFK-FH002C vaccine against SARS-CoV-2 in animals. The sample sizes were empirically estimated by considering the variations of the results and the statistical power needed while minimizing the number of animals. BALB/c mice were randomly assigned to groups of four to six mice to determine the antibody response induced by vaccine candidates. C57BL/6 mice were randomly assigned to groups of six to eight mice for determination of germinal center response and T cell response. Eight cynomolgus monkeys were randomly divided into four groups according to the same-sex ratio to determine safety and immunogenicity of StriFK-FH002C. For experiments in hamsters, groups of six or eight hamsters at a male to female ratio of 1:1 were challenged after being vaccinated, and then the protective effect of the vaccine was studied on the basis of indicators such as body weight, tissue viral RNA load, and lung pathology. Studies were not blinded. The number of technical replicates is described in the figure legends and varies among experiments.

### Constructs for recombinant protein expression

The codon-optimized RBD complementary DNA (cDNA) of SARS-CoV-2 (GenBank: MN908947.3) was obtained by primer annealing (data file S1). RBDTfd gene was produced by fusing with a trimeric foldon of the T4 phage head fibritin (Tfd) at the C terminus. The Tfd is a 27–amino acid peptide (YIPEAPRDGQAYVRKDGEWVLLSTFLG), which can facilitate protein trimerization (*47*). SARS-CoV-2 S1 and S2 subunit genes referring to the full-length spike glycoprotein gene of SARS-CoV-2 (GenBank: MN908947.3) were synthesized by General Biosystems Co. Ltd. StriFK is a trimeric spike glycoprotein ectodomain (amino acids 1 to 1207) containing three amino acid mutations in the furin-like cleavage site (RRAR to GSAS), similar to the noncleavable prefusion S0 reported by Sanchez-Felipe *et al. (**27**)*. Unlike the full-length S with mutation of the furin cleavage site and proline stabilizing mutations (S.PP) of Mercado *et al.* (*14*) and Corbett *et al.* (*48*), StriFK did not contain proline mutations for prefusion stabilization. All cDNAs were introduced with a C-terminal polyhistidine sequence, and codon optimization was performed for mammalian cell expression. These target genes were constructed into a modified PiggyBac transposon vector EIRBsMie using NEBuilder HiFi DNA Assembly Master Mix (E2621L, New England Biolabs) described previously (*49*).

### Experiment animals

BALB/c and C57BL/6 mice were purchased from Shanghai SLAC Laboratory Animal Co. Lakeview Golden (LVG) Syrian hamsters were purchased from Charles River Laboratories. Cynomolgus monkeys were from JOINN Laboratories Inc. All animal studies were carried out in strict accordance with the recommendations of the *Guide for the Care and Use of Laboratory Animals*. The mouse and hamster studies were conducted under the approval of the Institutional Animal Care and Use Committee of Xiamen University. The monkey experiments were conducted under the approval of the Institutional Animal Care and Use Committee of JOINN Laboratories Inc.

### Mouse immunization

For antibody response evaluation, BALB/c mice were maintained in a specific pathogen–free environment and immunized with various proteins at 1 μg per dose with Al001 or FH002C through intramuscular injection (150 μl per animal), following an immunization schedule of one priming dose at week 0 plus two boosters at weeks 2 and 4. Serum samples were collected at weeks 0, 1, 2, 3, 4, 5, and 6 via retro-orbital bleeding to measure the antibody titers. For cellular immune response analyses, C57BL/6 mice were immunized with 10 μg of StriFK with Al001 or FH002C adjuvants via intramuscular injection (150 μl per animal) on day 0 only or on days 0 and 21. Then, splenocytes and lymph node cells were collected on day 7 after the last immunization for IFN-γ ELISPOT or ICS measurements. To characterize germinal center responses, the T_fh_, GCB, and plasma cells in lymph nodes from immunized C57BL/6 mice at 1 week after single immunization were analyzed by flow cytometry.

### ELISPOT assay

Briefly, single-cell suspensions were obtained from mouse spleen (10^6^ cells per well) or lymph nodes (4 × 10^5^ cells per well) through grinding in cell strainers and were seeded in anti-mouse IFN-γ antibody precoated ELISPOT plates (Dakewe Biotech). Subsequently, cells were incubated with pooled peptides of SARS-CoV-2 spike (15-mer peptides with 11–amino acid overlap covering the entire spike protein; GenScript) and cultured at 37°C with 5% CO_2_ for 20 hours. The detection procedure was conducted according to the manufacturer's instructions. Spots were counted and analyzed by using CTL-ImmunoSpot S5 (Cellular Technology Limited). The numbers of IFN-γ–secreting cells were calculated by subtracting phosphate-buffered saline (PBS)–stimulated wells from spike peptide pool–stimulated wells.

### Anti-RBD, anti-spike, anti-S1, and anti-S2 IgG measurements

Microplates precoated with recombinant antigens of RBD, spike ectodomain, S1, or S2 were provided by Beijing Wantai. For detections, serially diluted (twofold) serum samples (100 μl per well) were added to the wells, and the plates were incubated at 37°C for 30 min, followed by washing with PBST buffer [20 mM PBS (pH7.4), 150 mM NaCl, and 0.05% Tween-20]. Then, horseradish peroxidase–conjugated anti-mouse IgG (Proteintech) for measurements of mouse sera, anti-human IgG (Thermo Fisher Scientific) for measurements of monkey sera, or anti-hamster IgG (Abcam) for measurements of hamster sera (100 μl per well) were added according to the species of samples. After a further 30-min incubation followed by washing with PBST buffer, tetramethylbenzidine (Wantai) chromogen solution (100 μl per well) was added to each well. Ten minutes later, the chromogen reaction was stopped by adding 50 μl of 2 M H_2_SO_4_, and optical density (OD)_450–630_ was measured. The IgG titer of each serum was defined as the dilution limit to achieve a positive result (greater than the mean plus 3 SDs of ODs of negative controls). Each plate contained five tests of negative control sera, and their ODs were used to determine the cutoff value. Representative data from technical replicates were performed at least twice for plotting.

### Pseudovirus neutralizing antibody titer measurement

Vesicular stomatitis virus (VSV)–based SARS-CoV-2 pseudotyping particles (VSVpp) were produced according to our previous study (*50*). The BHK21-hACE2 cells were used to perform the pseudovirus neutralization assay. Briefly, cells were preseeded in 96-well plates. Serially diluted (threefold gradient) samples were incubated with VSVpp inoculum for 1 hour. Next, the mixture was added into seeded BHK21-hACE2 cells. After a further incubation at 37°C containing 5% CO_2_ for 12 hours, fluorescence images were captured by Opera Phenix or Operetta CLS High-Content Analysis System (PerkinElmer). The counts of green fluorescent protein (GFP)–positive cells of each well were analyzed by the Columbus System (PerkinElmer). The neutralization titer of each sample was expressed as the maximum dilution fold (ID_50_) required to achieve infection inhibition by 50% (50% reduction of GFP-positive cell numbers compared to controls). The calculation of ID_50_ is based on the nonlinear fitting (variable slope) of the inhibition rate of the VSVpp by the serum after serial dilution by using GraphPad Prism 8. Representative data from technical replicates were performed at least twice for plotting.

### Receptor-blocking antibody titer measurement

A cell-based spike function-blocking test (CSBT) was used to characterize the receptor-blocking antibody titer in animal serum following the previously described procedure (*49*). Briefly, hACE2-mRuby3 293T (293T-ACE2iRb3) cells were constructed on the basis of human embryonic kidney (HEK) 293T cells [American Type Culture Collection (ATCC)] and were seeded in poly-d-lysine pretreated CellCarrier-96 Black plate (PerkinElmer) at 2 × 10^4^ cells per well and incubated overnight at 37°C with 5% CO_2_. Hamster or cynomolgus monkey sera were diluted in a twofold serial dilution with Dulbecco's modified Eagle’s medium (DMEM) containing 10% fetal bovine serum (FBS). Eleven microliters of Gamillus-fused SARS-CoV-2 spike trimer probe was mixed with 44 μl of the diluted sera to a final concentration of 2.5 nM. After removing half of the medium from the preseeded 293T-ACE2iRb3 cells, 50 μl of the mixture was added to the wells and incubated for 1 hour at 37°C with 5% CO_2_. Subsequently, cell images were captured by Opera Phenix High-Content Analysis System in confocal mode. Quantitative image analyses were performed by the Columbus System (PerkinElmer) following a previously described algorithm (*49*). CSBT activity (receptor-blocking antibody titers) of immunized sera was displayed as ID_50_, calculated by four-parameter logistic curve fitting in GraphPad Prism. Representative data from technical replicates were performed at least twice for plotting.

### SARS-CoV-2 virus challenges in hamsters

The SARS-CoV-2 virus (hCoV-19/Wuhan/AP8/2020) was isolated from a 32-year-old male patient in late January 2020, and the sequence was determined by Illumina MiSeq [Global Initiative on Sharing All Influenza Data (GISAID) accession number EPI_ISL_1655937]. Viral stocks were prepared in Vero cells (ATCC) with DMEM containing 2% FBS and 30 mM MgCl_2_. L-1-tosylamido-2-phenylethyl chloromethyl ketone-treated trypsin (5 μg/ml; Sigma-Aldrich) was added to keep the original features of the virus strain (*51*). Viruses were harvested, and the titers were determined by means of plaque assay in Vero cells as described below. Viral stocks (fourth passage, 1.36 × 10^6^ PFU/ml, 1 ml per stock) were stored in −80°C ultralow temperature freezer for animal study. Both intranasal challenge and direct-contact challenge of SARS-CoV-2 were used in the study. For the intranasal challenge, hamsters were inoculated with 1 × 10^4^ PFU of SARS-CoV-2 virus (diluted in 100 μl of PBS) through the intranasal route under anesthesia. For the contact challenge, virus-exposed hamsters (donors) were preinfected with inoculation of 1 × 10^4^ PFU of the SARS-CoV-2 virus through the intranasal route. One day later, each of the donors were transferred to a new cage and were cohoused with four vaccinated or unvaccinated control animals. Hamsters were given a limited amount of food daily with 7 g per 100 g of body weight to maintain a relatively stable weight. Weight changes and typical symptoms (piloerection, hunched back, and abdominal respiration) in hamsters were recorded daily beginning at the time of infection or cohousing. Hamsters were euthanized for tissue pathological and virological analyses on days 3, 5, or 7 after virus challenge. All virus challenge studies were performed in an animal biosafety level 3 (ABSL-3) facility.

### SARS-CoV-2 RNA quantification

Viral RNA concentrations in lung, trachea, and nasal turbinate from challenged hamsters were detected by quantitative reverse transcription polymerase chain reaction (qRT-PCR). Hamster tissue samples were homogenized with TissueLyser II (Qiagen) in 1 ml. RNA was extracted using the QIAamp Viral RNA Mini Kit (Qiagen) according to the manufacturer's instruction. Subsequently, viral RNA quantification was conducted using the SARS-CoV-2 RT-PCR Kit (WS-1248, Wantai). Representative data from technical replicates were performed at least twice for plotting.

### SARS-CoV-2 plaque assay

Plaque assays were performed in a biosafety level 3 (BSL-3) facility. Briefly, Vero cells were seeded in a six-well plate at 10^5^ cells per well. After overnight culture at 37°C with 5% CO_2_, the medium was removed, and the cells were washed twice with PBS. The supernatants of tissue lysates were serially 10-fold diluted with DMEM and were incubated with the cells for 1 hour at 37°C containing 5% CO_2_. Subsequently, the tissue lysate–containing medium was replaced with a mixture of DMEM containing 1% agar. After agar solidification, the cell plates were inverted and incubated at 37°C containing 5% CO_2_ for 3 days. Subsequently, cells were fixed with 2 ml of 4% formaldehyde for 2 hours at room temperature. The cells were further stained with 0.1% crystal violet for 20 min. The viral load was calculated on the basis of the count of plaques and the corresponding dilution factor.

### Histopathology

The lung tissues from challenged hamsters were fixed with 10% formalin for 48 hours and then embedded in paraffin and sectioned by microtome (Leica). Next, the fixed lung sections were stained with H&E. Immunohistochemistry staining for SARS-CoV-2 NP was used to evaluate viral antigen expression and distribution in lung tissues. A mouse anti–SARS-CoV-2 NP-specific antibody (15A7-1, developed in our laboratory) was used to detect virus by immunohistochemistry staining. Immunohistochemistry kits (#KIT-9730), Masson staining kits (#MST-8004), hematoxylin (#CTS-1096), and eosin (#CTS-4094) were purchased from Maxim Biotechnology. Whole-slide images of the lung sections were captured with the EVOS M7000 Images System (Thermo Fisher Scientific). Pathological lung lesions were scored in a blinded manner following a scoring standard. The pathological score included (i) alveolar septum thickening and consolidation; (ii) hemorrhage, exudation, pulmonary edema, and mucous; and (iii) recruitment and infiltration of inflammatory immune cells. For each lobe, a score was calculated in relation to severity (*52*): 0 indicates no pathological changes were observed, 1 indicates mild pathological change, 2 indicates moderate pathological change, 3 indicates severe pathological change, and 4 indicates very severe pathological change. For each animal, four independent lobes of the lung tissues were scored and the average score was used to reflect the pathological severity.

### Statistical analysis

All raw, individual-level data are presented in data file S2. Student’s *t* test or Mann-Whitney *U* test was used for the comparison of continuous variables between groups. A Kruskal-Wallis test was applied to analyze differences among more than two groups and corrected for multiple comparisons using Dunn's method. A paired Wilcoxon signed-rank test was applied to compare paired samples. A two-way analysis of variance was used to analyze the time-series observations for independent samples. A Pearson test and linear regression model were used for univariate correlation analysis. Statistical differences were considered to be significant for two-tailed *P* values of <0.05. Statistical analyses were conducted in GraphPad Prism 8 software.

## SUPPLEMENTARY MATERIALS

stm.sciencemag.org/cgi/content/full/13/606/eabg1143/DC1
Materials and Methods Figs. S1 to S13 Data files S1 and S2 References (*54*–*60*)

## Appendix

View/request a protocol for this paper from *Bio-protocol*.
